# Surgical site infection in critically ill patients with secondary and tertiary peritonitis: epidemiology, microbiology and influence in outcomes

**DOI:** 10.1186/s12879-015-1050-5

**Published:** 2015-07-30

**Authors:** Josep Ballus, Juan C. Lopez-Delgado, Joan Sabater-Riera, Xose L. Perez-Fernandez, A. J. Betbese, J. A. Roncal

**Affiliations:** Intensive Care Department, Hospital Universitari de Bellvitge, IDIBELL (Institut d’Investigació Biomèdica Bellvitge; Biomedical Investigation Institute of Bellvitge), C/Feixa Llarga s/n, 08907 L’Hospitalet de Llobregat, Barcelona Spain; Intensive Care Department, Hospital de la Santa Creu i Sant Pau, Barcelona, Spain; Universitat Autònoma de Barcelona, C/Sant Quintín 89, 08041 Barcelona, Spain

**Keywords:** Surgical site infection, Peritonitis, Intensive care, Outcomes

## Abstract

**Background:**

Surgical site infection (SSI) remains a significant problem in the postoperative period that can negatively affect clinical outcomes. Microbiology findings are typically similar to other nosocomial infections, with differences dependent on microbiology selection due to antibiotic pressure or the resident flora. However, this is poorly understood in the critical care setting. We therefore aimed to assess the incidence, epidemiology and microbiology of SSI and its association with outcomes in patients with severe peritonitis in the intensive care unit (ICU).

**Methods:**

We prospectively studied 305 consecutive patients admitted to our surgical ICU from 2010 to 2014 with a diagnosis of secondary or tertiary peritonitis. We collected the following data: SSI diagnosis, demographics, Acute Physiology and Chronic Health Evaluation (APACHE) II score, Simplified Acute Physiology Score (SAPS) II score, type of surgery, microbiology, antibiotic treatment and outcomes. Microbiological sampling was done by means of swabs.

**Results:**

We identified 269 episodes of SSI in 162 patients (53.1 %) aged 64.4 ± 14.3 years, of which 200 episodes occurred in men (64.6 %). The mean APACHE II and SAPS II scores were 19.7 ± 7.8 and 36.5 ± 16.1 respectively. The mean ICU and hospital stays were 19.8 ± 24.8 and 21.7 ± 30 days respectively. *Pseudomonas spp.* (*n* = 52, 19.3 %), *Escherichia coli* (*n* = 55, 20.4 %) and *Candida spp.* (*n* = 46, 17.1 %) were the most frequently isolated microorganisms, but gram-positive cocci (*n* = 80, 29.7 %) were also frequent. Microorganisms isolated from SSIs were associated with a higher incidence of antibiotic resistance (64.9 %) in ICU patients, but not with higher in-hospital mortality. However, patients who suffered from SSI had longer ICU admissions (odds ratio = 1.024, 95 % confidence interval 1.010–1.039, *P = 0.001*).

**Conclusions:**

The incidence of SSI in secondary or tertiary peritonitis requiring ICU admission is very high. Physicians may consider antibiotic-resistant pathogens, gram-positive cocci and fungi when choosing empiric antibiotic treatment for SSI, although more studies are needed to confirm our results due to the inherent limitations of the microbiological sampling with swabs performed in our research. The presence of SSI may be associated with prolonged ICU stays, but without any influence on overall mortality.

## Background

The skin is the main barrier against bacterial infection of internal tissues, and surgical wounds create a physical disruption to that barrier. The movement of bacteria across the skin barrier can lead to surgical site infections (SSI), one of the most frequent infectious complications of surgical procedures, with the potential risk for adverse outcomes [1]. SSI involves different inflammatory responses that range from low to high clinical significance [2], with that following abdominal surgery being a typical example associated with increased morbidity and mortality [2–5]. Worse still, SSI can spread to surrounding areas and vital deep structures, often requiring debridement or drainage [3, 4]. Consequently, the treatment of SSI leads to increased costs, especially when we consider the high number of surgical procedures and their complexity in a typical referral hospital [2–5].

Peritonitis, which is defined as inflammation of the serous membrane that covers the abdominal cavity and their organs, is classified into primary (spontaneous), secondary (process-related pathology in the visceral organs) and tertiary (persistent or recurrent after initial adequate surgical treatment). Secondary and tertiary peritonitis are associated with higher morbidity with mortality rates of 17–63 % [6, 7]. Tertiary peritonitis usually occurs in ICU settings at least 48–72 h following adequate treatment of secondary peritonitis, and has a mortality rate of 30–60 % [8].

Centers for Disease Control and Prevention (CDC) estimates that the risk of SSI associated with abdominal surgery ranges from approximately 2 to 8 %, depending on the type of surgery [2, 3, 9, 10]. SSI is classified into several categories: clean (2 %), clean-contaminated (3 %), contaminated (6 %) and dirty (7 %) [2, 11]. Stratification before surgery could help identify at-risk patients suitable for surveillance [3].

Despite the marked influence of SSI associated with severe peritonitis on public health and clinical practice, it has been poorly addressed in the literature, even in the ICU setting. This study therefore aimed to describe the incidence, epidemiology, microbiology and outcomes of SSI in patients admitted with secondary or tertiary peritonitis to the ICU of a tertiary referral hospital.

## Materials and methods

This prospective, observational study was carried out at from January 2010 to December 2014. At the time this study was performed, the Hospital Universitari de Bellvitge (HUB) was a tertiary hospital with 850 general care beds and 44 ICU beds. We included all consecutive patients from any type of abdominal surgery who required ICU admission beyond 72 h for secondary or tertiary peritonitis. All patients received standard preoperative hygiene care and antibiotic prophylaxis at anaesthetic induction consistent with our institutional protocols for elective and emergency surgery [4].

SSI was defined using the CDC definition [11] and diagnosis was by the responsible physicians, based on clinical criteria. Any purulent drainage from a surgical incision with signs of inflammation of the surrounding tissue was considered an SSI, whether microorganisms were isolated in cultures or not. The infection had to present at the surgical site within 30 days of surgery [2–4]. The study was approved by the Institutional Ethics Committee of our hospital (Comité d’ Ètica i Assajos Clínics de HUB (CEIC); Ethics and Clinical Assays Committee of HUB), and informed consent was waived due to the observational design.

In all patients, the decisions regarding ICU admission and treatment were made by the treating physician. Data were recorded from the medical registry of each patient in real time, using a standardised questionnaire, and collected in a database. The following information was recorded on admission: demographic data, medical history and comorbidities, surgical indication and type of surgery (elective or emergency), surgical technique, intraoperative variables (number of drains inserted), microbiologic findings, arterial lactate on admission and treatment characteristics. During their ICU stay, we also recorded the following: need for vasopressor drugs, mechanical ventilation and renal replacement therapy (RRT) and; any new microbiological findings, including the appropriateness, changes and resistance to antibiotic treatment. Illness severity was quantified with the Acute Physiology and Chronic Health Evaluation (APACHE) and Simplified Acute Physiology Score (SAPS) scoring systems during the first 24 h of ICU admission for all patients. After ICU discharge, follow-up was planned to collected data on in-hospital mortality and patients were followed until discharge from ICU or until resolution.

The surgical teams collaborated with ICU physicians to control the SSI, using simple washouts or serial debridements when appropriate. We obtained tissue samples and exudate samples, and direct needle aspiration was used when needed, in collaboration with the surgical team. The microbiological samples were obtained under conditions as sterile as possible in order to avoid colonizers of the superficial wound. The deepness of tissue sample was evaluated based on SSI characteristics. If necessary, drainage was performed, and any necrotic tissue was debrided and foreign material removed. Intensive irrigation with saline solution was employed when necessary to facilitate mechanical debridement [2]. We provided rational antibiotic therapy based on local guidelines and after consultation with an infectious disease physician.

For diagnosis purposes, microbiological samples were sent to the laboratory as swabs in culture media for semiquantitative aerobic and anaerobic cultures. To isolate anaerobes, specimens were inoculated into Columbia blood agar plates enriched with hemin and menadione, incubated in an anaerobic chamber at 37 °C, and specimens were Gram stained at 48 and 96 h for direct examination.

Statistical analysis was conducted using PASW Statistics 13.0 (SPSS Inc., Chicago, Illinois, USA). Continuous data are expressed as mean ± standard deviation and categorical data are expressed as percentages. Comparisons between groups with non-normal distributions were by two-sample *t*-tests or Mann–Whitney *U* tests after applying the one-sample Kolmogorov–Smirnov test. The *χ*^*2*^-test was used to evaluate categorical variables. Multivariable analysis was done to assess the influence of SSI and other SSI-related factors, such as the microbiology results, on mortality and outcomes. Odds ratios (ORs) and 95 % confidence intervals (CIs) are quoted as appropriate. A *P*-value of 0.05 was considered statistically significant in all cases.

## Results

**Fig. 1:**
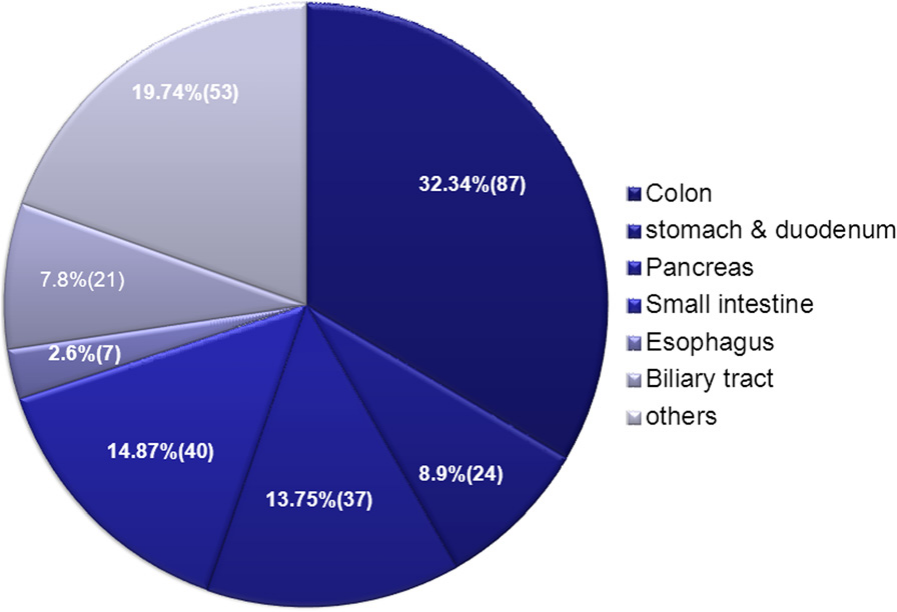
Types of surgery based on the anatomical location of the different identified SSI episodes

Of the 305 patients hospitalised for secondary or tertiary peritonitis in our ICU, we identified 269 episodes of SSI in 162 patients. The SSI rate of 53.1 % was higher in ICU compared with the rest of hospitalized patients who underwent major abdominal surgery (*n* = 193/935; 20.6 %) during the study period (*P* < 0.001). Patient characteristics, inflammatory response, type of surgery and outcomes are shown in Table 1. The mean duration of hospitalisation prior to surgery was 8.9 ± 2.5 days. Urgent abdominal surgery comprised 28 %–35 % of all abdominal surgeries performed at our hospital, showing a difference in the type of surgery in comparison with ICU patients (*P* = 0.01). The types of surgery (based on the anatomical location) of the different identified SSI episodes are shown in Fig. 1. All patients were monitored with a central venous catheter, arterial catheter and urinary catheter, and all patients were on vasoactive drugs or inotropic support.

**Table 1:** Patient characteristics, inflammatory response, type of surgery and outcomes

	All patients (*n* = 305)	Non-SSI subgroup (*n* = 143; 46.9 %)	SSI subgroup (*n* = 162; 53.1 %)	P
Age (years)	64.4 ± 14.3	63.5 ± 14.4	65.3 ± 13.8	0.27
Sex (male/ female)	200 (65.6 %)/105 (34.4 %)	86 (60.1 %)/57 (39.9 %)	114 (70.4 %)/48 (29.6 %)	0.07
APACHE II	19.7 ± 7.8	20.5 ± 8.7	18.95 ± 6.6	0.07
SAPS II	36.5 ± 17.1	37.9 ± 19.3	35.1 ± 13.9	0.13
Immunosuppression	36 (11.8 %)	18 (12.6 %)	18 (11.1 %)	0.72
Neutropenia on admission	14 (4.6 %)	7 (4.9 %)	7 (4.3 %)	1.00
HIV	37 (12.1 %)	19 (13.3 %)	18 (11.1 %)	0.60
Type of infection				
Secondary peritonitis	140 (45.9 %)	71 (49.6 %)	69 (42.6 %)	0.09
Tertiary peritonitis	165 (54.1 %)	72 (50.4 %)	93 (57.4 %)	0.023
Inflammatory response on admission				
Sepsis	27 (8.8 %)	11 (7.7 %)	16 (9.8 %)	0.85
Severe sepsis	32 (10.5 %)	15 (10.5 %)	17 (10.5 %)	1.00
Septic shock	204 (67 %)	89 (62.2 %)	115 (71.1 %)	0.006
Multi-organ failure	42 (13.7 %)	28 (19.5 %)	14 (8.6 %)	0.003
Surgery data				
Urgent surgery	276 (90.5 %)	125 (87.4 %)	151 (93.2 %)	0.11
Percutaneous surgery	41 (13.5 %)	17 (11.8 %)	24 (14.8 %)	0.10
Laparoscopic surgery	10 (3.3 %)	4 (2.8 %)	6 (3.7 %)	0.28
Laparotomy surgery	231 (75.7 %)	112 (78.5 %)	119 (73.5 %)	0.12
Combined surgery	23 (7.5 %)	10 (6.9 %)	13 (8 %)	0.14
Number of drainages on ICU admission	2 ± 1.7	1.2 ± 1.7	2.3 ± 1.8	0.01
Outcomes				
ARDS	121 (39.6 %)	55 (38.4 %)	66 (40.7 %)	0.85
On mechanical ventilation	279 (91.5 %)	130 (90.9 %)	149 (92 %)	0.83
Arterial lactate on admission (mmol · dL^−1^)	4.3 ± 3.3	4.1 ± 3.9	5.3 ± 2.5	0.57
RRT	77 (25.2 %)	40 (28 %)	37 (22.8 %)	0.35
Total parenteral Nutrition needs (any time)	251 (82.3 %)	113 (79 %)	138 (85.2 %)	0.17
ICU stay (days)	19.8 ± 24.8	12.9 ± 14.2	22.3 ± 32.9	0.002
Hospital stay (days)	21.7 ± 30	17.6 ± 35.3	25.4 ± 26.5	0.031
In-hospital mortality	115 (38 %)	64 (45.1 %)	110 (31.7 %)	0.018

Data are mean ± standard deviation or percentage

*SAPS* simplified acute physiology score, *APACHE* acute physiology and chronic health evaluation, *HIV* human immunodeficiency virus, *ARDS* acute respiratory distress syndrome, *RRT* renal replacement therapy, *ICU* intensive care unit

In the studied population, we identified 1181 positive culture isolates: 269 (22.7 %) were from SSIs, 486 (41.2 %) were from intra-abdominal abscesses, 251 (21.2 %) were from positive blood cultures (blood cultures) and 175 (14.9 %) were from other cultures. The microbiology results for isolates from SSI are shown in Table 2, with a preponderance of *Escherichia coli* (*n* = 55, 20.4 %) and *Pseudomonas aeruginosa* (*n* = 52, 19.3 %), while gram-positive cocci and *Candida spp.* were also frequent at rates of 29.7 % (*n* = 80) and 17.1 % (*n* = 46) respectively. Antibiotic resistance to two or more antibiotics occurred in 64.9 % (*n* = 174), with rates of Extended spectrum beta-lactamase-producing Enterobacteriaceae (*n* = 30) and *Pseudomonas aeruginosa* carbapenem-resistant (*n* = 32) of 11.1 % and 11.9 % respectively, but with low rates of typical multi-resistant microorganisms such as *Acinetobacter baumanii* (2.9 %, *n* = 8) and methicillin resistant *Staphylococcus aureus* (MRSA) (2.2 %, *n* = 6). The microorganisms isolated from intra-abdominal abscesses were the same of those isolated in SSI samples in the 20.9 % of the patients (*n* = 34) with similar rates of of multi-resistant bacteria. No relationship was established between the intra-abdominal abscesses and the occurrence of SSI based of clinical and surgical evaluation. Based on the culture antibiograms, 99.5 % of patients received appropriate antibiotic treatment. The most commonly used antibiotics are shown in Table 2, with the use of multiple, simultaneous or sequential antibiotics being used in 72.5 % of the cases (*n* = 195). A mean of 2.6 antibiotics was used per SSI, with treatment lasting 14 ± 8 days.

**Table 2:** Microbiology cultures and antibiotic therapy of surgical site infection

Isolated microorganisms	
*Escherichia coli*	20.4 % (*n* = 55)
*Pseudomonas aeruginosa*	19.3 % (*n* = 52)
*Candida albicans*	13.7 % (*n* = 37)
*Staphylococcus coagulase negative*	12 % (*n* = 32)
*Staphylococcus epidermidis*	7.4 % (*n* = 20)
*Enterococcus faecalis*	4.4 % (*n* = 12)
*Candida spp.* (Other than *C. albicans*)	3.3 % (*n* = 9)
*Acinetobacter baumanii*	2.9 % (*n* = 8)
*Klebsiella spp.*	2.6 % (*n* = 7)
*Proteus mirabilis*	
*Methicillin resistant Staphylococcus aureus*	2.2 % (*n* = 6)
*Staphylococcus aureus*	1.8 % (*n* = 5)
*Enterococcus faecium*	
*Enterobacter cloacae*	1.6 % (*n* = 4)
*Clostridium spp.*	
*Enterobacter aerogenes*	1.1 % (*n* = 3)
Other	
Antibiotic therapy	
Meropenem	28.6 % (*n* = 203)
Piperacillin-tazobactam	26.1 % (*n* = 185)
Teicoplanin	12.4 % (*n* = 89)
Cloxacillin	7.7 % (*n* = 55)
Fluconazole	7.7 % (*n* = 54)
Ertapenem	6.7 % (*n* = 48)
Imipenem-cilastatin	4.8 % (*n* = 34)
Other	6 % (*n* = 42)

Total mortality was 37.9 % (*n* = 116), from which 27.9 % (*n* = 85) correspond to ICU deaths. Although SSI was not associated with higher mortality in our population when confounders, such as variables that reflected disease severity, were included in the multivariate analysis, it was associated with a longer ICU stay (OR = 1.024, 95 % CI: 1.010–1.039; *P = 0.001*). Indeed, mortality was lower in the group with SSI (OR = 0.547, 95 % CI 0.323–0.924; *P = 0.024*). The need for RRT (OR = 3.358, 95 % CI: 1.619–6.966; *P = 0.001*) and prolonged ICU stay (OR = 1.071, 95 % CI: 1.046–1.097; *P < 0.001*) were associated with higher in-hospital mortality.

## Discussion

This study provides data on the incidence and microbiology of SSIs for a large cohort of critically ill patients admitted with secondary or tertiary peritonitis to a surgical ICU. It confirms that there was a high incidence of SSI in those patients. The main findings of our study were that SSI was associated with a prolonged ICU stay, but that it had little impact on the overall in-hospital mortality in our population.

The development of postoperative SSI is known to have been multifactorial, arising from a complex relationship between host and environmental factors [1, 2]. Host risk factors for SSI include morbid obesity, disease severity, advanced age, low blood-protein levels and malnutrition, diabetes, malignancy and sepsis, while other risk factors that include susceptibility include immunosuppression, smoking and having a distant infection site [10]. Pre-existing morbidity, the time of surgery and the type of SSI may also play key roles in the development of SSIs [10]. Thus, an increasingly elderly population with a greater number of comorbidities significantly increases the risk of developing an SSI [9, 12]. Critically ill patients represent an increasing proportion of the inpatient population that will undoubtedly lead to greater diagnostic and management challenges, especially given that most SSIs in the ICUs are nosocomial [9]. SSIs are most common in high-risk patients, with an incidence of about 11.7 % [12, 13].

Antibiotic prophylaxis reduces postoperative morbidity and length of hospital stay, which positively affects SSI-related costs [4]. Wounds with a risk of infection below 2 % do not generally require antibiotic prophylaxis, but notable exceptions include the placement of a prosthesis, cardiovascular surgery and neuro-surgery [14]. Up to 15 % of all elective surgical patients may develop an SSI, with rates as high as 30 % being common in contaminated or dirty surgical procedures [5]. In our study, the majority of procedures were considered dirty or contaminated, and many of the critically ill patients had markedly decreased serum protein concentrations. Together, these may ultimately explain our higher SSI rate. Our higher rates may also reflect the inherent risks of tertiary care institutions and the severity of our cohort. Therefore, our results may not be applicable to secondary and non-referral hospitals.

An SSI can increase hospital stay by about six days and can add 10–20 % to hospital costs, even leading to death; therefore, prevention and control should be an important component of healthcare quality control [3, 4]. SSIs may occur following any surgical incision, even after the use of minimally invasive techniques, so SSIs need to be reported through systematic monitoring programmes for nosocomial infection [15]. We showed that patients suffering from SSI in our cohort had longer ICU stays. However, we do not think this was simply a surrogate of higher illness severity in the SSI group because of the comparable severity scores between groups.

The dominant causative microorganisms and treatment options have changed over time. Today, most common pathogens are resistant to common antibiotics [16] with the need for a high index of suspicion, prompt operative intervention, appropriate antibiotic treatment and proper resuscitation [5]. Hypovolemia and hypothermia create peripheral vasoconstriction that leads to poor tissue perfusion, which facilitates the development of SSI in the presence of necrotic tissue, foreign bodies, hematomas and seromas [2]. The microbiology of intra-abdominal infections also varies depending on the source of infection, prior use of antibiotics, the site of infection and if it is community acquired or nosocomial.

Besides the host and wound factors, physicians should be aware of the increase in high-virulence species, such as *Staphylococcus aureus* and *Streptococcus pyogenes*. In addition, *Escherichia coli*, *Bacteroides fragilis* and other gram-negative, anaerobic pathogens are common in large bowel perforations [16]. Nosocomial intra-abdominal infections often involve microorganisms such as *Pseudomonas spp.*, *Enterococcus spp.* and fungi [5]. In our population, there was an increased presence of multidrug-resistant pathogens and fungal SSI rates when compared with other series [17, 18]. This could be explained by the higher antibiotic pressure, use of broad-spectrum antibiotics, illness severity and prolonged treatment periods. Indeed, concomitant treatment for peritonitis compounded matters. We also showed higher reliance on total parenteral nutrition (TPN) because enteral nutrition was poorly tolerated and we opted to initiate it early to avoid hypoalbuminemia, which is a risk factor for fungal infection and SSI in critically ill patients [19]. However, we concede that TPN is a risk factor for all types of fungal infection in ICU, especially among surgical patients [20].

The increasing trend to reduce hospital stays by implementing innovative surgical techniques (particularly minimally invasive and endoscopic procedures) makes it necessary to ensure that accurate measurement and monitoring of adverse events can take place after discharge. Without doing so, we cannot establish the real impact of SSI on morbidity and mortality [3, 9, 12]. Control measures with an emphasis on the education of healthcare professionals, such as frequent hand washing and the need to isolate patients with multi-resistant bacteria in cluster units, are necessary to reduce SSI rates [21]. Although the total elimination of SSI is not possible, a reduction in the rate of infection to a minimum should be achievable, even in critically ill patients [4].

Our study presents certain limitations. The most important is that we used for microbiological sampling a skin swabs instead of the “gold standard” for culture of skin, which are tissue biopsy or aspiration sampling of infected tissue. We could have obtained colonizer microorganisms that are not responsible for the infection and cultures may be misleading organisms in the deep tissue infection. Thus, our results should be considered cautiously and more studies are needed to confirm them. Secondly, this was a single-centre observational study and our results cannot be extrapolated to other less severely ill populations. Among the strengths of this study are the large sample size, the prospective entry of all data and the use of postoperative scores, which are not used in contemporary studies, even though their importance in risk stratification has been emphasised over recent decades. Furthermore, this investigation was conducted at a large tertiary referral hospital with a high level of complexity over a four-year period.

## Conclusions

In summary, the incidence of SSI was very high in patients with secondary or tertiary peritonitis requiring ICU admission. When they prescribe antibiotic therapy, physicians should consider that microorganisms isolated from patients with SSI are more likely to include multidrug-resistant pathogens, including *Pseudomonas spp.*, gram-positive cocci and fungi, although more studies are needed to confirm our results due to the inherent limitations of the microbiological sampling with skin swabs performed in our research. Despite the presence of an SSI may be associated with prolonged ICU stays, we did not find any effect on the in-hospital mortality in our population.

## Abbreviations

SSI: Surgical site infection
ICU: Intensive care unit
APACHE: Acute physiology and chronic health evaluation
SAPS: Simplified acute physiology score
RRT: Renal replacement therapy
TPN: Total parenteral nutrition
ARDS: Acute respiratory distress syndrome in adults
